# Quantitative Determination and Validation of Four Ketones in *Salvia miltiorrhiza* Bunge Using Quantitative Proton Nuclear Magnetic Resonance Spectroscopy

**DOI:** 10.3390/molecules25092043

**Published:** 2020-04-28

**Authors:** Yuanyuan Li, Zhuoni Hou, Feng Su, Jipeng Chen, Xiaodan Zhang, Ling Xu, Dongfeng Yang, Zongsuo Liang

**Affiliations:** 1 The Key Laboratory of Plant Secondary Metabolism and Regulation of Zhejiang Province, College of Life Sciences and Medicine, Zhejiang Sci-Tech University, Hangzhou 310018, China; 2 Key Laboratory for Green Pharmaceutical Technologies and Related Equipment of Ministry of Education, College of Pharmaceutical Sciences, Zhejiang University of Technology, 18 Chao Wang Road, Hangzhou 310014, China

**Keywords:** qNMR, *Salvia Miltiorrhiza* Bunge, tanshinone I, tanshinone IIA, dihydrotanshinone, cryptotanshinone

## Abstract

*Salvia mltiorrhiza* Bunge (SMB) is native to China, whose dried root has been used as medicine. A few chromatographic- or spectrometric-based methods have already been used to analyze the lipid-soluble components in SMB. However, the methodology of qNMR on the extracts of fresh SMB root has not been verified so far. The purpose of this study was to establish a fast and simple method to quantify the tanshinone I, tanshinone IIA, dihydrotanshinone, and cryptotanshinone in fresh *Salvia Miltiorrhiza* Bunge root without any pre-purification steps using ^1^H-NMR spectroscopy. The process is as follows: first, 70% methanol aqueous extracts of fresh *Salvia Miltiorrhiza* Bunge roots were quantitatively analyzed for tanshinone I, tanshinone IIA, dihydrotanshinone, and cryptotanshinone using ^1^H-NMR spectroscopy. Different internal standards were tested and the validated method was compared with HPLC. 3,4,5-trichloropyridine was chosen as the internal standard. Twelve samples of *Salvia Miltiorrhiza* Bunge were quantitatively analyzed by qNMR and HPLC respectively. Then, the results were analyzed by chemometric approaches. This NMR method offers a fast, stable, and accurate analysis of four ketones: tanshinone I, tanshinone IIA, dihydrotanshinone, and cryptotanshinone in fresh roots of *Salvia Miltiorrhiza* Bunge.

## 1. Introduction

*Salvia miltiorrhiza* Bunge (SMB, *danshen* in Chinese), belonging to the Labiate family [1,2], is an important traditional Chinese herbal plant with a long history as a medicine as well as a health food [3]. The active ingredients of SMB can be divided into two major groups: lipid-soluble (lipophilic) tanshinones including tanshinone I (Tan I), tanshinone IIA (Tan IIA), tanshinone IIB (Tan IIB), dihydrotanshinone (DTS) and cryptotanshinone (CTS) [4,5], and water-soluble (hydrophilic) phenolic acids such as danshensu (DSU), caffeic acid (CA), rosmarinic acid (RA), salvianolic acid A (Sal A) and salvianolic acid B (Sal B) [6].

Modern pharmacological studies have shown that tanshinones have many pharmacological activities such as (1) antioxidation: CTS has antioxidant in vivo and in vitro pharmacological activities [7,8]; Tan IIA can effectively inhibit the interaction of intracellular lipid peroxidation products with DNA, eliminate the lipid free radicals produced by the lipid peroxidation pathway in the mitochondrial membrane of the myocardium, thus protecting the respiration of mitochondria [9]. (2) Anti-atherosclerosis: Tan IIA can promote cholesterol efflux, ameliorate lipid accumulation in macrophages, and reduce the development of aortic atherosclerosis [10]. (3) Antibacterial: Gram-positive bacteria can be significantly inhibited by tanshinones such as CTS and DTS. (4) Antitumor: DTS, Tan I, Tan IIA and CTS in blood can inhibit growth and induce the apoptosis of malignant tumor cells [11]; Tan I exhibits anti-cancer activity on various human cancers which significantly inhibits osteosarcoma (OS) cancer cell proliferation, migration, invasion and induced cell apoptosis in vitro [12]; CTS shows significant antitumor effects by inducing apoptosis of tumor cells [13]; DTS exerts an effective antitumor effect by inhibiting tumor cell proliferation and promoting tumor cell apoptosis [14]; Tan IIA inhibits cell proliferation and induces cell differentiation by affecting the cell cycle; on the other hand, it increases the expression of bax/bcl-2 protein by the Fas pathway to induce apoptosis. In addition, Tan IIA can also choose to activate members of the Caspase family to exert its anti-cancer effect [15].

**Figure 1:**
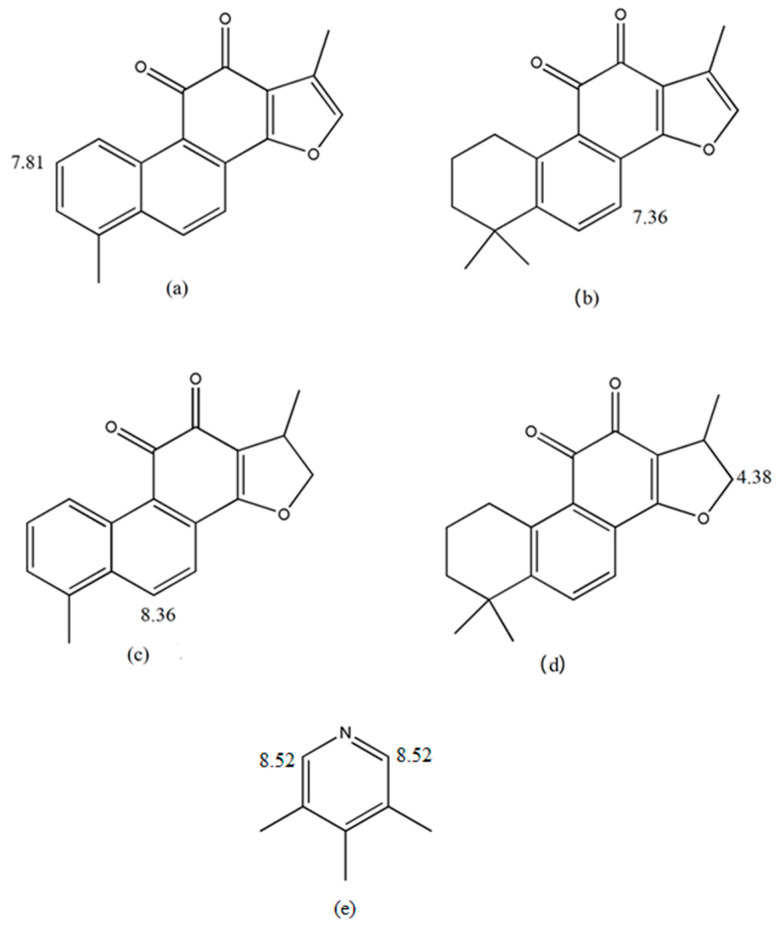
Chemical structures of Tan I (**a**), Tan IIA (**b**), DTS (**c**), CTS (**d**) and 3,4,5-trichloropyridine (**e**) in SMB. (The numbers in the figure represent the chemical shifts of the marked peaks of the NMR spectrum of each compound, unit is ppm).

With the increasing research on regulation pathways of tanshinones, it is necessary to develop an accurate and sensitive quantitative method for content determination of the tanshinones. A few analytical techniques including HPLC, UHPLC-Q-Exactive Orbitrap mass spectrometry, LC-MS/MS and NMR have been successfully applied for lipid-soluble ketone determinations [16,17,18,19]. However, to the best of our knowledge, the methodology of quantitative NMR on the extract of SMB fresh roots has not been verified so far. qNMR method is very efficient for the simultaneous detection and identification of several metabolites in crude extracts or samples [20,21,22]. Compared with traditional quantitative methods, qNMR spectroscopy has the following advantages: (1) no calibration standard of the analyte is needed; (2) only an inexpensive internal standard is needed; (3) high selectivity can be achieved under appropriate acquisition conditions; (4) more than one analyte can be determined at one time; (5) reduced measuring time. Thus, establishing a reliable qNMR-based method for measuring lipid-soluble ketone content in SMB is desirable. Furthermore, a quality evaluation model of SMB may be established by combining NMR profiles with chemometrics. Here, we quantified the levels of IS as well as Tan I, Tan IIA, CTS and DTS from fresh SMB root samples using ^1^H-NMR (Figure 1). Specifically, 12 batches of SMB were profiled using ^1^H-NMR. Next, a principal components analysis (PCA) and cluster analysis were conducted to determine the sample correlation. To validate our results, the levels of four ketones were determined using HPLC. We found that the ^1^H-NMR technique provided a reliable means of quantifying SMB-derived ketones and may be used as a supplementary tool for HPLC-based analyses.

## 2. Results and Discussion

### 2.1. Quantitative Analysis of SMB Using ^1^H-qNMR

The sample solutions were optimized to obtain the best separation and stability for all the integrated signals in the ^1^H-NMR spectrogram. Quantitation was performed using an NMR sample-tube adapter at 298 K. Prior to all the measurements, the analyte and IS were qualitatively analyzed using routine ^1^H experiments to determine the longest spin-lattice relaxation time (usually at least 5 times the T1, thus the optimized relaxation delay of 25 s was obtained). The 3,4,5-trichloropyridine was chosen as the IS due to its good solubility and stability.

Two basic requirements for an IS should be met.; one is that the signal of the IS and the target signal from the analyte should not overlap, and the other is that the resonance of the IS and the selected compound should not occur in crowded spectral regions. 3,4,5-trichloropyridine was chosen as an IS for the quantitative analysis.

### 2.2. Validation of qNMR Analytical Method

#### 2.2.1. Specificity and Selectivity

**Figure 2:**
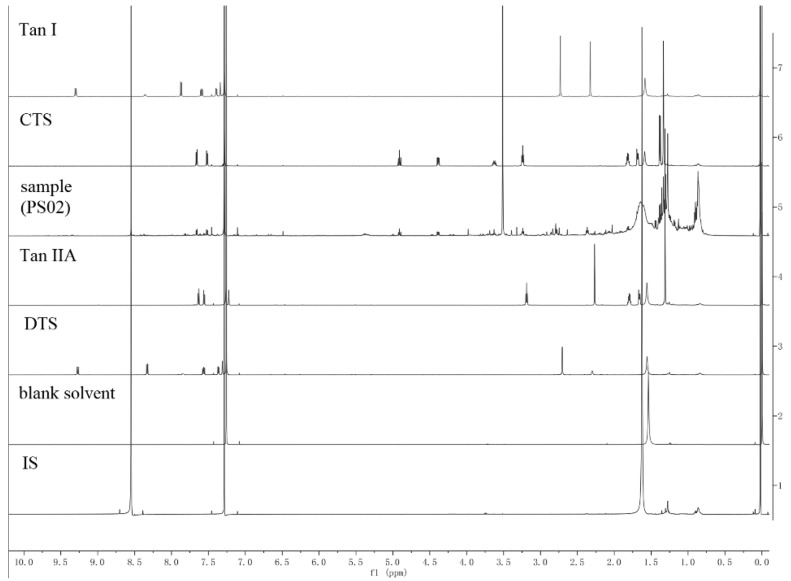
^1^H-NMR spectra of Tan Ⅰ, CTS, sample (PS02), Tan ⅡA, DTS, blank solvent and IS.

**Figure 3:**
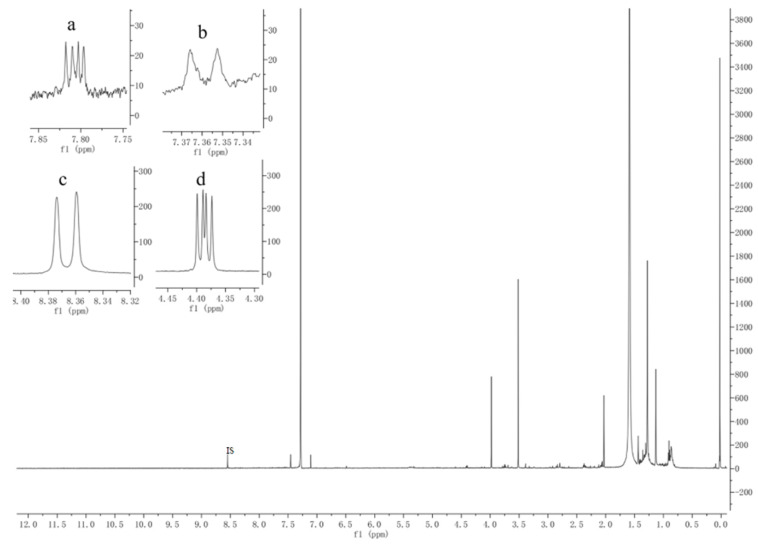
Representative 1H-NMR spectra for Tan I (**a**), Tan IIA (**b**), DTS (**c**) and CTS (**d**) with IS in the full range of 0 to 12.0 ppm. (Material is PS02, a, b, c, and d respectively represent the local magnifications of the four signal peaks).

Specificity and selectivity are key prerequisites that must be evaluated to avoid possible interference from other components in the sample solution. We thus compared the ^1^H-NMR spectra of the standard references and sample solution with IS, and IS and solvent individually (Figure 2). We found that the resonances assigned to these protons are quite separated from the others, such as to have a good integration without interferences (Figure 3), compared with the data reported in the literature [19], signals for Tan Ⅰ at 7.81 ppm (dd,1H), Tan ⅡA at 7.36 ppm (d,1H), DTS at 8.36 ppm (d,1H), CTS at 4.38 ppm (dd,1H) were selected as the quantitation signals.

#### 2.2.2. Linearity, LOD and LOQ

The intensity of the response signal is directly proportional to the amount of nuclei, as described in Equation (1). Consequently, the linearity regression yielded a good correlation coefficient (r^2^ > 0.985). The concentration ratios of the four references ranged between 0.02 and 0.18 mg/mL (CTS), 0.02 and 0.24 mg/mL (Tan I), 0.03 and 0.33 mg/mL (Tan IIA) and 0.02 and 0.24 mg/mL (DTS), respectively.

The limit of detection (LOD) presents the lowest detectable analyte concentration, whilst the limit of quantitation (LOQ) represents the lowest quantifiable analyte concentration. These are two fundamental elements of method validation defining the limitations of an analytical method. In qNMR, the LOD and LOQ cannot be determined by means of SNR (signal noise ratio) as the NMR response signals are Lorentzian lines. Hence, the LOD and LOQ were determined using the standard deviation of the response *σ* and the slope *S* of a calibration curve obtained in the linearity study by Equations (1) and (2):

(1) $$LOD=\frac{3.3\sigma }{s}$$

(2) $$LOQ=\frac{10\sigma }{s}$$

The results for linearity, LOD and LOQ are shown in Table 1.

#### 2.2.3. Stability

To determine the optimal time window between sample collection and analysis, it is crucial to conduct a sample stability test. A solution with an RSD (relative standard deviation) value of less than 3.0% is considered stable. We analyzed the stability of the sample solution with IS of PS07 at room temperature (~25 °C) at 0, 6, 12, 18 and 24 h intervals and determined their RSD values. The content difference and RSD values of the sample solution are shown in Table 2.

#### 2.2.4. Accuracy

The accuracy of an analytical method is determined by measuring the correlation of theoretical value and the value found. To study the accuracy of our method, we carried out a recovery study by adding a known amount of Tan I, Tan IIA, DTS and CTS into the SMB extraction system, respectively. Each concentration level was repeated in triplicate. The accuracy was estimated by measuring the mean recovery and relative standard deviation (RSD). The recovery was determined by comparing the calculated value of added reference material and true value according to Equation (3):

(3) $$Recovery=\frac{m_{x}-m_{o}}{m_{s}}\times 100\%$$

where *m_x_* is the calculated mass of analyte after reference addition, *m_o_* is the calculated mass of original analyte before reference addition and *m_s_* is the true mass of the reference addition. The results are summarized in Table 3.

#### 2.2.5. Precision and Intermediate Precision

ICH guidelines recommend that precision is assessed using six repeated determinations. In this study, the intermediate precision was evaluated by a second analyst on an alternative probe (5 mm PABBO 600S3 BBF-H-D-05 Z SP (Z114607)). The calculated content of each analyte and statistical result were shown in Table 4. There were no significant differences in precision and intermediate precision between the results.

#### 2.2.6. Robustness

The robustness of an analytical procedure is measured in terms of its capacity to remain unaffected by small, but deliberate variations in method parameters listed in the procedure documentation, which indicates suitability during normal routine usage. In this study, PS07 was used to test for robustness. Three parameters were varied independently: (1) number of scans; (2) relaxation delay; (3) acquisition time. We observed that our method was largely unaffected by variations in all of the tested parameters (Table 5).

### 2.3. Quantitative Analysis of SMB by HPLC

A set of standard solutions of Tanshinone IIA (10.9, 109, 163, 217, 326 μg/mL), Dihydrotanshinone (7.9, 23.6, 118, 157, 236 μg/mL), Tanshinone I (7.7, 23.2, 116, 155, 232 μg/mL) and Cryptotanshinone (4.5, 13.6, 68, 90.7, 136 μg/mL) were used to prepare the calibration curve. The content of the four ketones in 12 batches of crude SMB extract determined by HPLC is shown in Table 6.

### 2.4. Real Sample Determination

#### 2.4.1. Quantitative NMR Analysis of Real Samples

PS01-PS12 were also analyzed using qNMR, and the results obtained from the two methods were compared. We observed no significant differences between the results obtained using HPLC and qNMR (Table 6). Therefore, qNMR represents an alternative method of quantifying compounds within complex matrices.

#### 2.4.2. Sample Representation

**Figure 4:**
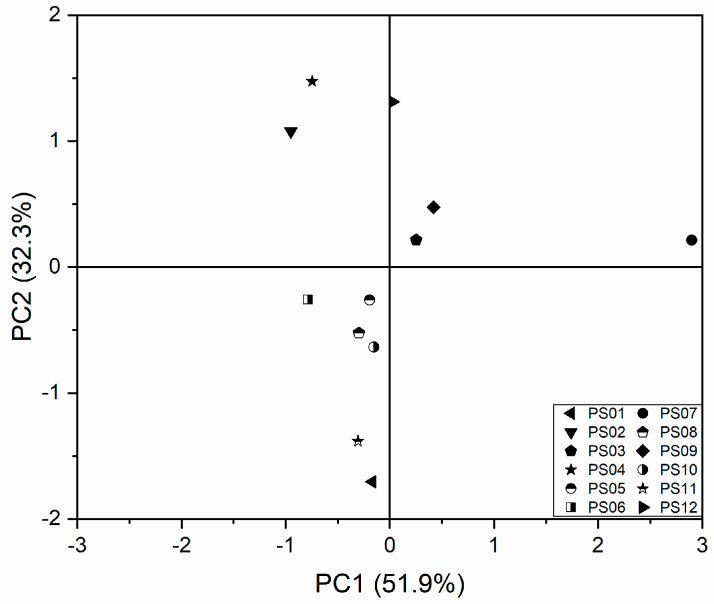
PCA core plots for 600 MHz ^1^H-NMR spectra of SMB extracts from 12 collection areas.

PCA is often used to reveal the clustering behavior of samples. We observed different clustering behaviors for sample extracts obtained from different areas (PC1: 51.9%, PC2: 32.3%). As shown in the PCA scores plot (Figure 4), most samples clustered to the middle of the plot. However, PS07 clustered different from the other samples. The samples of PS08 and PS10 are relatively concentrated.

**Figure 5:**
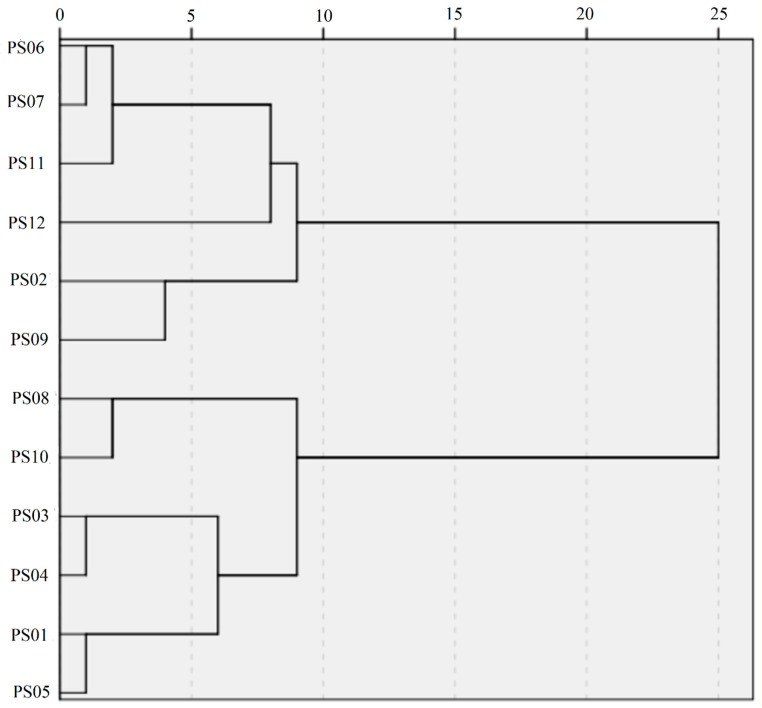
Cluster analysis of the 12 batches of SMB samples.

To further investigate our observation, the cluster analysis, which measures the distances or correlation between objects [23,24,25,26], was carried out. Consistent with the PCA results, we found that PS07 clustered the furthest away from the other samples (Figure 5). In the dendrogram, PS08 and PS10 were belong to the same node.

**Figure 6:**
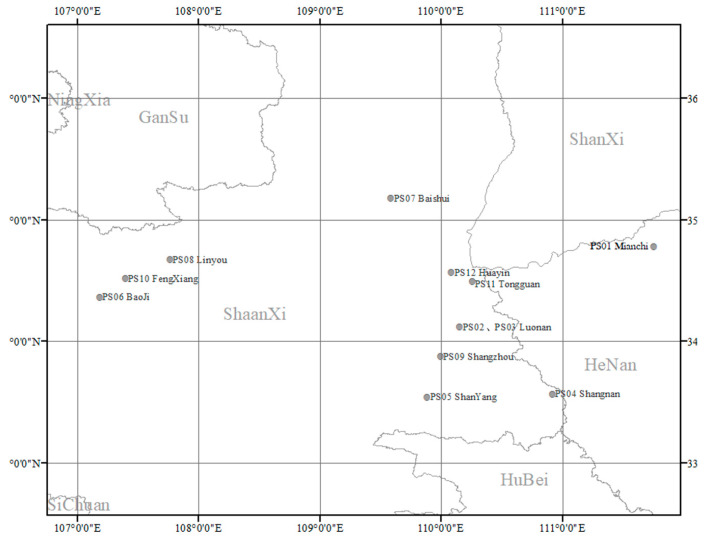
Geographic information of PS01–PS12.

Our results were consistent with the geographic information of these samples (Figure 6). Among them, the collection area of PS07 has the highest latitude, while the collection areas of PS08 and PS10 have similar latitudes, which indicates that the latitude of the collection area may have some impact on the cluster results of some sample.

## 3. Materials and Methods

### 3.1. Plant Material and Reagents

Fresh SMB roots (PS01–PS12) were obtained from Mianchi (Henan, China), Fengxiang, Baoji, Shangnan, Luonan, Linyou, Baishui, Shanyang, Shangzhou, Tongguan, Huayin (Shaanxi, China) from Shaanxi Tasly Pharmaceutical Co. Ltd. Reference standards Tan I (HPLC ≥ 98%), Tan IIA (HPLC ≥ 98%), CTS (HPLC ≥ 98%) and DTS (HPLC ≥ 98%) were obtained from the Shanghai Yuanye Biotechnology Co. Ltd. (China). Chloroform-D (CDCl_3_) were purchased from Qingdao Asfirst Science Co. Ltd. (D, 99.8%, Qingdao, China), 3,4,5-trichloropyridine were purchased from Shanghai Aladdin Bio-chemical Technology Co. Ltd. (GC, 98%, Shanghai, China), in which CDCl_3_ was used as solvent and 3,4,5-trichloropyridine was used as the internal standard (IS). Methanol and acetonitrile of chromatographic grade (TEDIA) were used for extraction and HPLC analysis. Analytical grade phosphoric acid (Damao Chemical Reagent Factory, Tianjin, China) was used for the HPLC analysis. The pure water used in this study was obtained using a Barnstead TII Super Pure Water System (Thermo Fisher Scientific, Boston, MA, USA).

### 3.2. Instruments and Parameters

Fresh SMB roots samples were milled using a Q-250B1mill (Shanghai, China). A KH5200DE ultrasonic bath (Nanjing, China) was used for extraction.

The samples for qNMR analysis were measured on a 600 MHz Avance III HD spectrometer with a TXI probe at 298K (Bruker Corporation, Faellanden, Switzerland). ^1^H-NMR experimental parameters were shown as follows: zg30 pulse sequence with 32 scans of 32 K data points in a spectral width of 12019.2 (20 ppm), acquisition time 5.0 s, relaxation delay 25 s. All of the data processing was performed by using MestReNova11.0 and SPSS21.0: first, we use MestReNova’s superposition function to superimpose the internal standard, solvent, sample, and 4 standards, and select the appropriate signal peak. Then, the sample is integrated according to the selected signal peak, and the content of the four components in the sample is calculated by combining with the standard curve. After the above process is completed, SPSS is used for principal component analysis and cluster analysis.

### 3.3. Preparation of Sample Solution

Approximately 2.0 g of the SMB root powder was added into 20 mL 70% methanol aqueous solution and settled for 12 h at room temperature, then extracted for 45 min in an ultrasonic bath. The supernatant was cooled to room temperature and filtered. Next, 1500 μL of filtered liquor was transferred into a clean Eppendorf tube and nitrogen at 35 °C. The 600 μL of CDCl_3_ (containing 0.03% *v*/*v* TMS) was then added to dissolve the sample for NMR analysis. The NMR sample tube was assembled for analysis at 298 K and sealed prior to the ^1^H-NMR measurement. For HPLC analysis, the filter liquor was directly injected into the HPLC system. All of the extractions and subsequent NMR measurements were performed in triplicate.

### 3.4. Internal Standards

The extracted sample PS02 with 600 μL CDCl_3_, containing 0.13 mg 3,4,5-trichloropyridine was analyzed as IS.

### 3.5. Quantitative NMR Analysis

The most important fundamental relation of qNMR is the signal response (integrated signal area) *I*_x_ in a spectrum that is directly proportional to the number of nuclei *N*_x_ generating the corresponding resonance line [27,28]:

(4) $$I_{x}=K_{s}N_{x}$$

*K*_s_ is an unknown spectrometer constant, which is a constant for all resonance lines in the same ^1^H single-pulse NMR spectrum. Accordingly, the determination of the relative area ratios *I*_x_/*I*_y_ is the most efficient way to obtain quantitative results by using Equation (5) when *K*_s_ cancels for the ratio:

(5) $$\frac{I_{x}}{I_{y}}=\frac{N_{x}}{N_{y}}$$

For the purity determination of a substance, an internal standard with known purity is needed. Based on Equation (5), the component purity can be calculated from the NMR intensity via Equations (6) and (7):

(6) $$W_{x}=\frac{I_{x}\times N_{Std}\times M_{x}\times m_{Std}}{I_{Std\;}\times N_{x}\times M_{Std}}$$

(7) $$P_{x}=\frac{I_{x}\times N_{Std\;}\times M_{x}\times m_{Std}\times P_{Std}}{I_{Std}\times N_{x}\times M_{Std}\times m}$$

*W_x_* and *P_x_* represent the mass and purity of the analyte. *M_x_* and *M_Std_* are the molar masses of the analyte and the standard (3,4,5-trichloropyridine: 182.44 g/mol). *m* is the weighed mass of the investigated sample. *m_Std_* and *P_Std_* are the weighed mass and the purity (99.5%) of the standard. *N_Std_* and *I_Std_* correspond to the number of protons for the standard (in this experiment is 2) and the integrated signal area of a typical NMR line (which was 2 in this experiment). *N_x_* and *I_x_* correspond to the number of protons for the analyte ^1^H.

### 3.6. HPLC Analysis

The qNMR results were verified using HPLC. The HPLC experiments were performed using an Agilent 1260 HPLC system (Agilent, Palo Alto, CA, USA) equipped with a Diode-array detector and a Sunfire C_18_ column (5 μm, 250 mm × 4.6 mm i.d., Waters, Milford, MA, USA). The mobile phase consisted of 0.02% phosphoric acid aqueous solution (A) and acetonitrile (B), programmed as follows: 0–10 min, 5–20% B; 10–15 min, 20–25% B; 20–25 min, 25–20% B; 25–28 min, 20–30% B; 28–40 min, 30% B; 40–45 min, 30–45% B; 45–58 min, 45–58% B; 58–67 min, 58–50% B; 67–70 min, 50–60% B; 70–80 min, 60–65% B; 80–85 min, 65–95% B; 85–95 min, 95–95% B; 85–96 min, 95–5% B. The detection wavelength was set at 270 nm and flow rate was 1 mL/min. The content of DTS, Tan IIA, Tan I and CTS in each sample was determined the external standard method and the calibration curve of the corresponding standards.

### 3.7. Chemometrics Methods

Principal component analysis (PCA) and hierarchical cluster analysis were used to analyze the NMR data.

## 4. Conclusions

A selective and accurate qNMR method was established for quantitatively determining and validating four lipid-soluble ketones in SMB extracts. The results of the analysis of linearity, precision, stability, accuracy, LOD and LOQ demonstrated that ^1^H-NMR can be used to accurately determine the content of ketones. We found that the results generated using the qNMR method were consistent with those obtained using HPLC analysis. Whilst HPLC took more than 60 min to complete, qNMR took only 19 min for each sample. Due to the good linearity between IS and the references, qNMR can be performed without any standard references. Although future studies will be required to maximize its application potential, NMR profiles can be combined with chemometric approaches to give insights into the relationship between the quality, properties and geographic origins of plant-based materials. In summary, qNMR presents a rapid and effective method of analyzing various plant extracts and their by-products.

## Figures and Tables

**Table 1:** Linearity, LOD and LOQ of four lipid-soluble ketones.

Reference	Calibration Curves	Range (mg/mL)	r^2^	LOD (mg/mL)	LOQ (mg/mL)
Cryptotanshinone	Y = 0.926X − 0.006	0.02–0.18	0.999	0.02	0.05
Tanshinone I	Y = 1.231X − 0.003	0.02–0.24	0.997	0.04	0.11
Tanshinone IIA	Y = 0.715X − 0.018	0.03–0.33	0.992	0.09	0.28
Dihydrotanshinone	Y = 0.740X − 0.001	0.02–0.24	0.985	0.04	0.11

**Table 2:** Results for the stability study.

	Tanshinone I		Tanshinone IIA		Dihydrotanshinone		Cryptotanshinone	
Times (h)	Content (mg/g)	Difference (mg/g)	Content (mg/g)	Difference (mg/g)	Content (mg/g)	Difference (mg/g)	Content (mg/g)	Difference (mg/g)
0	0.65	NA	1.75	NA	0.37	NA	0.98	NA
6	0.69	0.04	1.79	0.04	0.40	0.03	0.97	0.01
12	0.67	0.02	1.76	0.01	0.39	0.02	1.02	0.04
18	0.67	0.02	1.70	0.05	0.39	0.02	1.02	0.04
24	0.67	0.02	1.77	0.02	0.39	0.02	1.02	0.04
RSD%	2.11		1.92		2.82		2.49	

**Table 3:** Accuracy test results.

Compounds	Tanshinone I	Tanshinone IIA	Dihydrotanshinone	Cryptotanshinone
Level 1 (mean/%, *n* = 3)	65.16	74.09	121.08	71.13
RSD/%, *n* = 3	0.98	0.98	0.52	0.80
Level 2 (mean/%, *n* = 3)	96.30	85.37	102.27	123.21
RSD/%, *n* = 3	0.89	1.08	1.29	0.59
Level 3 (mean/%, *n* = 3)	85.85	114.01	145.81	93.50
RSD/%, *n* = 3	0.53	0.39	0.19	0.72

**Table 4:** Precision and intermediate precision test results ^a.^

Study	Precision				Intermediate precision			
	Tanshinone I (mg/g)	Tanshinone IIA (mg/g)	Dihydrotanshinone (mg/g)	Cryptotanshinone (mg/g)	Tanshinone I (mg/g)	Tanshinone IIA (mg/g)	Dihydrotanshinone (mg/g)	Cryptotanshinone (mg/g)
1	0.63	1.72	0.36	0.95	0.68	1.76	0.38	1.00
2	0.66	1.73	0.38	1.00	0.65	1.75	0.38	0.98
3	0.65	1.71	0.38	1.02	0.69	1.72	0.40	0.98
4	0.63	1.72	0.36	0.95	0.69	1.76	0.40	0.99
5	0.67	1.76	0.38	0.98	0.66	1.73	0.38	1.00
6	0.65	1.75	0.37	0.98	0.67	1.77	0.39	1.02
RSD%	2.47	1.12	2.65	2.81	2.81	2.43	1.11	1.52

^a^ Precision studies were determined on 5 mm ID probe, and intermediate precision studies were tested on an alternative NMR probe (5 mm PABBO 600S3 BBF-H-D-05 Z SP (Z114607)), by a different analyst on different days.

**Table 5:** Results for robustness study.

Parameters		Tanshinone I (mg/g)	Tanshinone IIA (mg/g)	Dihydrotanshinone (mg/g)	Cryptotanshinone (mg/g)
Number of scans	6	0.65	1.75	0.37	1.08
	32	0.67	1.70	0.39	1.11
	48	0.65	1.74	0.38	1.06
	64	0.67	1.77	0.39	1.06
RSD%		1.75	1.69	2.50	2.19
Relaxing delay	15	0.67	1.70	0.39	1.02
	20	0.65	1.68	0.38	0.99
	25	0.67	1.70	0.39	1.02
	30	0.67	1.70	0.39	1.02
RSD%		1.50	0.59	1.29	1.48
Acquisition time	2s	0.65	1.67	0.37	0.98
	4s	0.67	1.64	0.39	0.93
	6s	0.64	1.65	0.36	0.95
RSD%		2.24	0.92	2.63	2.64

**Table 6:** Assay (%) of four lipid-soluble ketones acid in the 12 different origins crude extract of SMB determined by the 1H NMR and HPLC methods.

Samples	Methods	Cryptotanshinone		Tanshinone I		Tanshinone IIA		Dihydrotanshinone	
		Content (mg/g) ± SD	RSD%	Content (mg/g) ± SD	RSD%	Content (mg/g) ± SD	RSD%	Content (mg/g) ± SD	RSD%
PS01	HPLC	0.29 ± 0.00	0.01	0.33 ± 0.00	0.01	1.21 ± 0.00	0.00	0.10 ± 0.00	0.01
	NMR	0.29 ± 0.03	0.10	0.30 ± 0.05	0.17	1.19 ± 0.08	0.06	0.15 ± 0.03	0.18
PS02	HPLC	0.68 ± 0.00	0.00	0.44 ± 0.01	0.01	1.39 ± 0.00	0.00	0.42 ± 0.00	0.00
	NMR	0.66 ± 0.03	0.05	0.40 ± 0.00	0.03	1.36 ± 0.09	0.06	0.44 ± 0.01	0.02
PS03	HPLC	0.61 ± 0.01	0.01	0.46 ± 0.00	0.01	1.51 ± 0.00	0.00	0.40 ± 0.00	0.01
	NMR	0.69 ± 0.02	0.02	0.49 ± 0.03	0.06	1.51 ± 0.03	0.02	0.37 ± 0.02	0.04
PS04	HPLC	0.68 ± 0.00	0.01	0.54 ± 0.00	0.01	1.62 ± 0.00	0.00	0.47 ± 0.00	0.00
	NMR	0.67 ± 0.01	0.02	0.54 ± 0.02	0.04	1.60 ± 0.10	0.06	0.45 ± 0.01	0.01
PS05	HPLC	0.31 ± 0.00	0.01	0.30 ± 0.00	0.01	0.87 ± 0.00	0.00	0.23 ± 0.00	0.00
	NMR	0.37 ± 0.03	0.09	0.32 ± 0.02	0.06	0.88 ± 0.03	0.03	0.29 ± 0.03	0.11
PS06	HPLC	0.31 ± 0.00	0.00	0.30 ± 0.00	0.00	0.95 ± 0.00	0.00	0.14 ± 0.00	0.01
	NMR	0.30 ± 0.01	0.05	0.32 ± 0.02	0.05	1.01 ± 0.09	0.09	0.12 ± 0.02	0.18
PS07	HPLC	1.06 ± 0.01	0.01	0.67 ± 0.00	0.00	1.84 ± 0.01	0.00	0.49 ± 0.00	0.00
	NMR	1.03 ± 0.06	0.05	0.66 ± 0.01	0.01	1.83 ± 0.07	0.04	0.42 ± 0.06	0.15
PS08	HPLC	0.37 ± 0.00	0.00	0.38 ± 0.00	0.01	1.34 ± 0.24	0.00	0.20 ± 0.00	0.00
	NMR	0.38 ± 0.02	0.05	0.38 ± 0.02	0.04	1.25 ± 0.00	0.18	0.23 ± 0.01	0.06
PS09	HPLC	0.82 ± 0.01	0.00	0.59 ± 0.00	0.00	1.99 ± 0.00	0.00	0.34 ± 0.00	0.00
	NMR	0.76 ± 0.03	0.04	0.57 ± 0.04	0.06	1.91 ± 0.09	0.04	0.36 ± 0.02	0.06
PS10	HPLC	0.38 ± 0.00	0.01	0.45 ± 0.01	0.01	1.38 ± 0.00	0.00	0.21 ± 0.00	0.01
	NMR	0.39 ± 0.07	0.19	0.42 ± 0.02	0.04	1.24 ± 0.07	0.05	0.23 ± 0.01	0.05
PS11	HPLC	0.63 ± 0.00	0.00	0.38 ± 0.00	0.01	1.36 ± 0.00	0.00	0.34 ± 0.03	0.00
	NMR	0.68 ± 0.03	0.05	0.38 ± 0.03	0.08	1.19 ± 0.12	0.10	0.30 ± 0.00	0.09
PS12	HPLC	1.08 ± 0.01	0.00	0.50 ± 0.00	0.00	2.02 ± 0.00	0.00	0.49 ± 0.01	0.00
	NMR	1.18 ± 0.02	0.01	0.50 ± 0.03	0.05	1.97 ± 0.03	0.02	0.47 ± 0.01	0.01
